# Eurasian Red Squirrels (*Sciurus vulgaris*) in Wildlife Rehabilitation Centres: Causes of Admission, Outcomes, and Seasonal Trends in the Czech Republic

**DOI:** 10.3390/ani16152411

**Published:** 2026-08-05

**Authors:** Gabriela Kadlecová, Anežka Hubáčková, Eva Voslářová

**Affiliations:** Department of Animal Protection and Welfare and Veterinary Public Health, Faculty of Veterinary Hygiene and Ecology, University of Veterinary Sciences Brno, 612 42 Brno, Czech Republic; hubackovaanezka@seznam.cz (A.H.); voslarovae@vfu.cz (E.V.)

**Keywords:** juveniles, anthropogenic activity, transport, injury

## Abstract

The Eurasian red squirrel (*Sciurus vulgaris*) is considered an indicator species in relation to forest ecosystems as well as the coexistence of wildlife and humans in urbanized environments. This study focused on the intake of Eurasian red squirrels into rehabilitation centres in the Czech Republic between 2010 and 2020. A total of 5365 individuals were admitted, with an increasing trend in admissions over this period (rSp = 0.9727, *p* < 0.05). The highest number of squirrels was admitted in 2020 (*p* < 0.05), while the highest monthly number was recorded in April (*p* < 0.05). The most frequently admitted cases were orphaned juveniles (57.93%), followed by juveniles from threatened nests (17.78%). A substantial proportion of Eurasian red squirrels were released back into the wild (52.69%); however, a considerable number died (28.15%) or were euthanized (3.37%), with the proportions of mortality, release, and other outcomes varying according to the reason for admission. Mortality occurred most frequently in cases of vehicle collisions, whereas the highest proportion of animals released back into the wild was observed among juveniles from threatened nests. Juveniles from threatened nests also spent the longest time in rehabilitation centres prior to release, together with individuals admitted following animal attacks (*p* > 0.05; median lengths of stay: 66.0 and 70.5 days, respectively). Data from rehabilitation centres can contribute to more effective care for injured or weakened wild animals and improved preparedness of these centres during the busiest period of the year, improved prognostic evaluation and targeted field interventions aimed at reducing negative anthropogenic impacts on wildlife.

## Simple Summary

The Eurasian red squirrel (*Sciurus vulgaris*) is an important indicator species of forest ecosystems and urban wildlife–human interactions. This study analysed admissions of Eurasian red squirrels to rehabilitation centres in the Czech Republic between 2010 and 2020. In total, 5365 individuals were admitted, with a significant increasing trend over time and a peak in spring (April). Most admitted animals were orphaned juveniles, followed by juveniles from threatened nests and injured adults. Over half of the individuals were successfully released back into the wild, while about one third died and a small proportion were euthanized, with mortality depending on admission cause. Juveniles and traffic-involved individuals showed the highest mortality rates. These results highlight the impact of human activities on Eurasian red squirrel populations and demonstrate the importance of rehabilitation centres not only for wildlife care, but also for improving public awareness and supporting better wildlife management and conservation strategies.

## 1. Introduction

The Eurasian red squirrel (*Sciurus vulgaris*) inhabits large parts of Europe [1,2,3]. It is a relatively adaptable arboreal rodent capable of living not only in forests, its natural habitat, but also in urban parks and other urbanized areas [4]. Given its mode of life, this species plays an important role in maintaining ecosystems, particularly forest stands. It can therefore serve as a suitable indicator of environmental condition and the degree of habitat fragmentation [5], as in some countries these habitats are undergoing a very rapid decline [6]. Interventions in forest ecosystems, such as tree felling or the use of modern technologies in forestry, significantly alter the composition of forest stands and transform original habitats. Species that rely on extensive forests for their survival, foraging, and reproduction are dependent on these habitats, and their transformation represents a substantial threat to them. The Eurasian red squirrel is not classified as a threatened species according to the International Union for Conservation of Nature. However, an estimated 10–20% of all squirrel species are highly threatened or at risk of extinction [7]. In addition to the rapid decline of forested areas, the Eurasian red squirrel is affected by negative pressures associated with increasing urbanisation, expanding transport infrastructure, and the introduction of non-native species [8]. Road construction contributes to further habitat fragmentation and hinders the natural movement of animals, which may become victims of collisions with vehicles [9]. In the case of invasive species, competition with the highly successful eastern grey squirrel (*Sciurus carolinensis*) was documented, with its negative impacts described particularly in the United Kingdom and in some other parts of Europe [10]. In addition, this species is known to carry squirrelpox virus, which is often fatal to the native Eurasian red squirrel and represents one of the key factors contributing to its decline in invaded areas [11]. The eastern grey squirrel is a highly resilient and adaptive species. It was classified by the International Union for Conservation of Nature as one of the world’s 100 worst invasive species due to its significant negative impacts in areas of introduction [7].

Although increasing urbanisation and the expansion of human settlements represent a significant barrier to the survival of Eurasian red squirrels in urban environments [12], the surrounding landscape influences squirrel movements. However, the landscape influence appears to have only a negligible effect on gene flow between populations [13]. Studies even indicate that cities may, conversely, provide favourable conditions for them [14], and therefore squirrel population densities in urban areas are often higher than in rural environments [12]. High-quality and well-preserved urban green spaces may represent an important refuge for Eurasian red squirrels, as they provide not only sufficient food resources but also suitable nesting sites [14].

As Eurasian red squirrels often live in close proximity to human settlements, they are not only more frequently exposed to anthropogenic factors, but also more often come to the attention of the public, which increases the likelihood of finding individuals in distress. These factors further highlight the need for effective protection of this species at both international and national levels, and it has therefore become the subject of considerable interest in wildlife conservation, with a range of conservation measures being implemented. These include, for example, the control of populations of the invasive eastern grey squirrel [15], the reintroduction of the Eurasian red squirrel into its natural habitat [16], and measures aimed at reducing road mortality [17]. In the Czech Republic, the Eurasian red squirrel is protected by national legislation and is listed among specially protected species [18], which ensures the protection of both individuals and their habitats. One form of wildlife protection is the activity of rehabilitation centres, which operate across individual countries and contribute to the care of weakened or injured individuals with the aim of returning them to the wild [19,20,21].

The public can play an important role in detecting animals in danger, particularly in synanthropic or partially synanthropic species such as the Eurasian red squirrel. Despite the high number of individuals admitted to rehabilitation centres worldwide, there is still relatively limited published information on the number of treated animals, the quality of care provided, and especially their subsequent fate after release. Data obtained from rehabilitation centres may nevertheless represent a valuable source of information on the health status of free-living wildlife populations and may also contribute to understanding the spread of diseases, including those with potential zoonotic implications [22].

The aim of this study was to evaluate the number of Eurasian red squirrels admitted to rehabilitation centres in the Czech Republic between 2010 and 2020, including the reasons for their admission and removal from records. At the same time, the study aimed to assess seasonal trends in admissions, the sex of admitted individuals, and the mortality of Eurasian red squirrels in rehabilitation centres, and thus to evaluate the importance of rehabilitation centres in the care of this species.

## 2. Materials and Methods

Data on Eurasian red squirrel (*Sciurus vulgaris*) individuals admitted to 37 rehabilitation centres in the Czech Republic between 2010 and 2020 were obtained from the database of the Ministry of the Environment of the Czech Republic. The database included, for each individual, the reason for admission, the date of admission, and outcome of rehabilitation. For a subset of individuals, information on the place of finding, place of release, sex, age, and body mass was also available.

Based on the available official data, the number of individuals admitted to rehabilitation centres in the Czech Republic between 2010 and 2020 was compiled. Subsequently, the seasonal dynamics of admissions throughout the calendar year were evaluated. A total of 27 individuals were excluded from this analysis because the exact date of admission was unknown and only the year was available.

For individuals with recorded sex (*n* = 2252), the numbers of admitted squirrels were compared between sexes. The numbers of admitted individuals were further analysed according to the reason for admission and the reason for removal from rehabilitation centre records. The proportions of individual outcome categories were also evaluated for each reason for admission.

The length of stay in rehabilitation centres was assessed for individuals subsequently released into the wild (*n* = 2826), with comparisons made among the different reasons for admission. Length of stay was defined as the interval between the date of admission and the date of discharge from the rehabilitation centre. Animals admitted and released on the same day were assigned a length of stay of 0.5 days to account for the time required for clinical examination.

For analytical purposes, individual cases were classified into categories according to the reason for admission (Table 1) and the reason for removal from records (Table 2).

Statistical analysis was performed using Unistat 6.5 for Excel (Unistat Ltd., London, UK). To assess the number of Eurasian red squirrels admitted per calendar year over the study period, data normality was first tested. Based on the results of this test, Spearman’s rank correlation coefficient was subsequently used to evaluate the trend (increasing or decreasing) in the number of admitted individuals between 2010 and 2020.

Chi-square tests with Yates’ correction were used to compare frequencies according to years of admissions, sex, reasons for admission, and reasons for removal from records, including an analysis of outcomes in relation to admission causes. For variables containing multiple categories, pairwise comparisons were performed between all categories using chi-square tests with Yates’ correction. Superscript letters indicating statistical groupings were assigned based on the resulting *p*-values (*p* < 0.05); categories sharing the same letter did not differ significantly, whereas categories with different letters showed significant differences. These comparisons were used as descriptive analyses to characterize the distribution of admission categories and outcomes within the dataset. Differences in the length of stay were compared by Kruskal–Wallis ANOVA. Statistical significance was defined as α = 0.05.

## 3. Results

**Figure 1:**
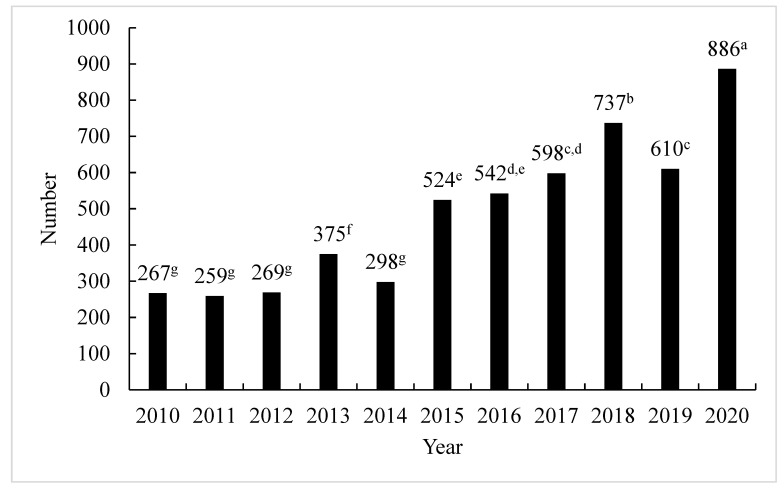
Number of Eurasian red squirrels admitted to rehabilitation centres in the Czech Republic between 2010 and 2020 (*n* = 5365). ^a–g^ Different letters indicate statistically significant differences (*p* < 0.05) in the numbers of animals admitted in different years.

Overall, 5365 Eurasian red squirrels were admitted to 37 rehabilitation centres in the Czech Republic between 2010 and 2020 (Figure 1). Spearman’s correlation test confirmed an increasing trend in the number of admitted Eurasian red squirrels over the study period (rSp = 0.9727, *p* < 0.05). In 2010, 2011, 2012, and 2014, the numbers of admitted individuals were comparable (*p* > 0.05) and were the lowest recorded throughout the study period, whereas the highest numbers were recorded in 2020 (*p* < 0.05), with 886 (16.51%) admitted individuals.

**Figure 2:**
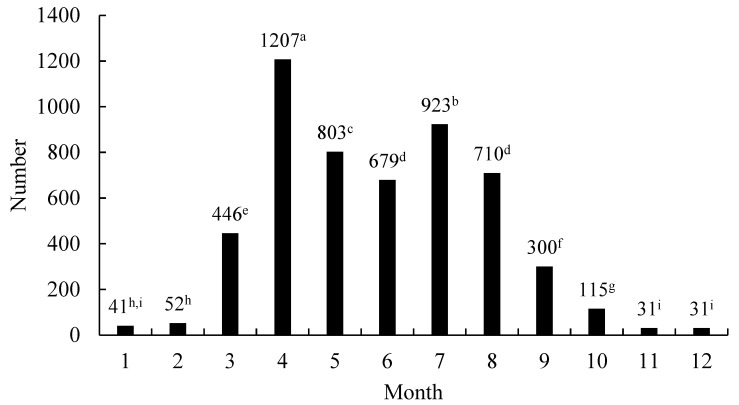
Number of Eurasian red squirrels admitted to rehabilitation centres in the Czech Republic between 2010 and 2020 by month of admission (*n* = 5338). ^a–i^ Different letters indicate statistically significant differences (*p* < 0.05) in the numbers of animals admitted in different months.

The highest number of individuals was admitted during the spring and summer months, with April being the dominant month, accounting for 1207 individuals (22.61%; Figure 2). The lowest numbers of individuals were admitted in November and December. 

Significantly more (Table 3; χ^2^(1) = 17.91, *p* < 0.001) male individuals than female individuals were admitted to rehabilitation centres in the Czech Republic between 2010 and 2020.

The predominant admission category was orphaned juveniles (*n* = 3108; 57.93%; χ^2^(9) = 14,954.80, *p* < 0.001; Table 4). Orphaned juveniles represented a significantly more frequent admission category compared with all other admission categories (*p* < 0.05). The second most frequent category was juveniles from threatened nests, representing 954 individuals (17.78%) of the total number of admitted Eurasian red squirrels.

The most frequent reason for removal of Eurasian red squirrels from rehabilitation centre records was release back into the wild (Table 5), which concerned 2827 individuals, representing 52.69% of all cases. The second most common reason was unassisted death of squirrels during care in the rehabilitation centre, recorded in 1510 individuals (28.15%).

Table 6 presents a comparison between outcomes in individuals admitted to rehabilitation centres according to their reason for admission. The results showed that the highest proportions of released squirrels were recorded in the categories of juveniles from threatened nests (68.97% in the category of admissions) and unnecessarily captured individuals (61.29%). In contrast, unassisted death was the most frequent outcome among squirrels admitted following vehicle collisions (72.88%). For the categories of infection, exhaustion, and unnecessarily captured individuals, no significant differences were found between the proportions of squirrels released back into the wild and those that died unassisted (*p* > 0.05).

**Figure 3:**
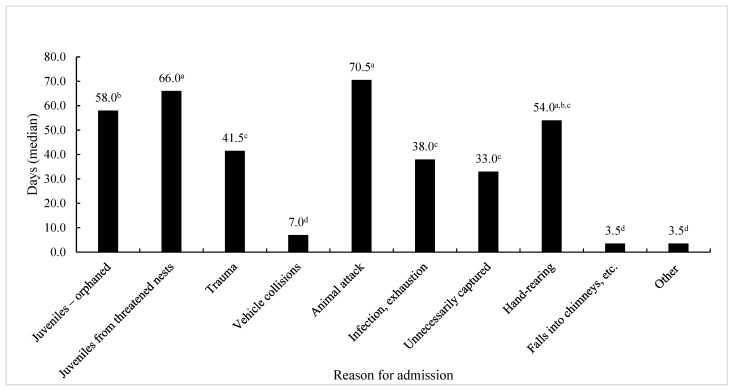
Length of stay (median number of days) of released Eurasian red squirrels admitted to rehabilitation centres in the Czech Republic between 2010 and 2020, according to the reason for admission (*n* = 2826). ^a–d^ Different letters indicate statistically significant differences (*p* < 0.05) in the median length of stay of released animals among the different reasons for admission.

Squirrels admitted following animal attacks and juveniles from threatened nests had the longest stays in rehabilitation centres before being released back into the wild, with median lengths of stay of 70.5 and 66 days, respectively, with no significant difference between these categories (*p* > 0.05; Figure 3). In contrast, individuals admitted after falls into chimneys and similar structures and those admitted following vehicle collisions had the shortest stays before release, with median lengths of stay of 3.5 and 7 days, respectively, with no significant difference between these categories (*p* > 0.05).

## 4. Discussion

The increasing trend in admissions of Eurasian red squirrels to rehabilitation centres in the Czech Republic is consistent with trends reported for other species or groups of animals [23,24,25]. The overall increase observed during the study period was not substantially influenced by interannual fluctuations, including the deviation in 2019, when 610 Eurasian red squirrels were admitted, i.e., fewer than in the previous year 2018 (737 individuals admitted). Although the highest number of squirrel admissions was recorded in 2020, this pattern should be interpreted in the context of the overall long-term increasing trend rather than as a distinct change restricted to a single year. The long-term increase in admissions is more likely to be explained as a consequence of growing public awareness regarding appropriate procedures when encountering wild animals, as well as improving education in this field [26]. In the case of the Eurasian red squirrel, an additional important factor may be its positive perception by the public, who often regard it as a cute and beneficial animal [27], which may increase people’s willingness to intervene and contact rehabilitation centres.

The highest number of individuals was admitted during the spring and summer months, with April being the dominant month, accounting for 1207 individuals. In contrast, a marked decrease in admissions was observed during the autumn months (*p* < 0.05), with the lowest numbers recorded in November and December (31 individuals in both months). This seasonal pattern has also been described in several other studies focusing on the admission of wild animals to rehabilitation centres, where repeated peaks in spring and summer and minimum values in the winter period have been observed [21,28]. This pronounced seasonal pattern is mainly associated with the reproductive period, during which offspring are born, are particularly vulnerable, and more frequently come into contact with humans, thereby increasing the likelihood of their detection and admission to rehabilitation centres [21]. The lower number of admissions in autumn and winter may, on the other hand, be related to reduced animal activity and a lower level of interaction with human populations [29]. Seasonal admission patterns are also influenced by the Eurasian red squirrel’s winter torpor [30]. During winter, the species undergoes periods of torpor, interrupted by episodes of activity during which squirrels leave their shelters and search for food [31]. Seasonal differences in squirrel activity may also vary between urban parks and forest environments, with higher winter activity recorded in urban areas, likely due to the year-round availability of supplementary food, whereas activity patterns in forest environments differ [32]. These differences suggest that environment and resource availability may substantially influence squirrel behaviour throughout the year [33]. Seasonal fluctuations in the number of admitted individuals also have important practical implications for the functioning of rehabilitation centres, as higher workloads in spring and summer require increased personnel, spatial, and material capacities, whereas these demands are lower during the winter period.

Sex distribution showed a significant difference (*p* < 0.05), with males being admitted more frequently than females. However, the effect size was small and sex was not recorded for a substantial proportion of individuals. This difference may be partially explained by sex-specific differences in spatial behaviour in the Eurasian red squirrel. Squirrels expand their home range in response to low food availability [34]. Males typically occupy larger territories than females, and home range size is positively correlated with male body size and dominance [35]. However, higher mobility and greater spatial activity of males may increase the likelihood of encounters with anthropogenic threats, such as more frequent road crossings, and thus a higher probability of vehicle collisions, resulting in their more frequent admission to rehabilitation centres [36]. Nevertheless, this interpretation should be treated with caution given that the study population represents rehabilitation admissions rather than the wild population, and sex was unknown for a proportion of individuals.

The most frequently admitted category of individuals to rehabilitation centres in the Czech Republic consisted of orphaned juvenile Eurasian red squirrels (57.93% of the total number of admitted individuals), highlighting the importance of juvenile admissions in wildlife rehabilitation centres [21,37]. However, not all juveniles admitted by members of the public are truly orphaned, as in many cases they are incorrectly removed from their natural environment due to a misinterpretation of the temporary absence of the mother. This issue is particularly common in species in which females leave their offspring unattended for short periods [38], which may create a perception of abandonment among lay observers. In the case of the Eurasian red squirrel, this situation is further intensified by the attractiveness of juveniles and their apparent vulnerability, which may lead to an over-sensitive response by observers and subsequent unnecessary intervention. Rehabilitation centres therefore not only provide care for animals but also fulfil an important educational role [39]. They help inform the public that it is necessary to distinguish between truly orphaned individuals and juveniles that are only temporarily left by their mother. In this way, they contribute to reducing unnecessary captures and interventions in free-living populations. Juveniles are often very difficult to rear, and mortality in rehabilitation centres is high [40]. This reduces their chances of reaching adulthood in the wild, where juvenile mortality is generally high even without human intervention, for example due to starvation [41]. Nevertheless, juvenile Eurasian red squirrels may in certain periods show a real increase in mortality or the presence of specific impairments preventing their survival, particularly during the transition to independence, when they are more exposed to predation or to the necessity of learning to survive on their own [42]. Loss of the mother, for example due to traffic [43], during this period may have fatal consequences for juvenile survival, as care is provided almost exclusively by the female.

The second most frequent group (17.78%) consisted of juveniles from damaged or threatened nests, which may be destroyed in connection with forestry activities or climatic influences [44,45]. Although the Eurasian red squirrel is a protected species in the Czech Republic, in practice, the presence of nests is not always thoroughly verified before logging operations are initiated. This is particularly true on private lands, where supervision is often more limited. A more pronounced increase in admitted individuals around 2020 can also be associated with extensive timber harvesting due to bark beetle outbreak, which may have led to increased disturbance of nesting habitats and subsequently to a higher number of juveniles transferred to rehabilitation centres.

Common causes of traumatic injuries in free-living animals include interactions with domestic animals, with small herbivores in particular frequently being victims of attacks by domestic cats [19]. Species inhabiting urban and suburban environments are especially vulnerable to such attacks, and predation may lead to severe injuries requiring subsequent care [46]. Injuries caused by domestic animals likely represent an important cause of trauma in wildlife; however, species-level identification of the attacking animal was not available in the present study. Although previous studies have reported both dogs and cats as potential sources of wildlife injuries [47], such species-specific comparisons cannot be made based on the present dataset. Currently, there is limited evidence that predation by domestic animals is a limiting factor for Eurasian red squirrel populations in urban areas. Nevertheless, it may represent a significant risk to individual health [14]. In rehabilitation centres in the Czech Republic, 259 bitten squirrels were admitted, representing 4.83% of all admissions. Mortality in this group was among the highest, together with animals admitted following vehicle collisions or infection/exhaustion, indicating the severity of these conditions. It is therefore evident that the rehabilitation of bitten individuals is mostly unsuccessful, particularly due to the frequent presence of internal injuries.

The release rate of rehabilitated or hand-reared Eurasian red squirrels is comparable to, or slightly higher than, that reported in other studies, with 52.69% of individuals being released. This relatively high proportion of successful returns to the wild may be one indicator of effective care and the professional expertise of rehabilitation centre staff. However, successful reintroduction of individuals into the wild is strongly dependent on their initial health status at admission to rehabilitation centres. Animals admitted in poor condition have a lower probability of successful recovery and release, and also require a longer period of hospitalisation than individuals admitted in good condition [48]. In free-living wildlife, not only the nature of the primary impairment but also the time until detection and initiation of treatment plays an important role. To be released back into the wild, an individual must satisfy several criteria beyond being able to move and being in good health. It must be capable of recognising, obtaining, and processing food typical of its species, as well as recognising and avoiding predators, or actively defending itself against them. It must also be able to secure and defend an appropriate shelter, perform normal seasonal movements, and interact with conspecifics [49]. Only a proportion of individuals survive after release due to natural mortality, for example due to predation. Therefore, it is important to maximise their chances by preserving natural behavioural patterns. It should be noted that release from rehabilitation centres does not automatically indicate successful long-term survival in the wild. As post-release monitoring was not available in this study, our conclusions are limited to short-term outcomes recorded during rehabilitation.

Eurasian red squirrels in rehabilitation centres had a higher frequency of unassisted death than euthanasia across all admission categories, with the exception of hand-rearing and falls into chimneys and similar structures; however, this finding contradicts some other studies. For example, Mullineaux and Pawson [21] in the United Kingdom reported that animals were more frequently euthanized (37.2%) than found dead (19.2%). These findings may serve as a basis for more targeted staff training and more efficient allocation of financial resources and conservation efforts, particularly towards case types with the highest probability of successful release. Death typically occurs as a consequence of unsuccessful treatment of injuries, disease, or other impairments that were the direct reason for admission to the rehabilitation centre [50], although stress related to the injury itself, as well as confinement in an unfamiliar environment and human presence, may also play a role [51]. The highest proportion of unassisted deaths was observed in squirrels involved in vehicle collisions (72.88% of all individuals admitted following vehicle collisions), whereas the highest proportion of euthanasia was recorded in cases of trauma (21.71% of the respective admission category). Road traffic has also been shown to be a major cause of mortality in Eurasian red squirrels in the wild [41]. A study conducted in the Czech Republic by Borkovcová et al. [52], focusing on wildlife road mortality, reported that the Eurasian red squirrel was the third most frequently killed small mammal species on Czech roads. In cases where a diagnosis with a poor prognosis is established, euthanasia performed by a veterinarian may be preferred. However, this remains a relatively sensitive topic that often generates public debate. Euthanasia is an important component of rehabilitation centre procedures to prevent pain and suffering in wild animals, particularly in sick or injured individuals. In some cases, euthanasia may appear to be the most appropriate option because the individual’s welfare is compromised by severe suffering, pain, or a minimal chance of survival. Nevertheless, decision-making is not always straightforward. In certain cases, a period of observation, further clinical examination, assessment, or laboratory testing may be necessary. However, the priority should always be the preservation of the individual’s welfare, even if this involves ending suffering through controlled euthanasia.

The length of stay in rehabilitation centres varied depending on the reason for admission. Squirrels admitted following animal attacks and juveniles from threatened nests required the longest rehabilitation periods prior to release. In cases of animal attacks, injuries are likely to be severe. As shown above, they are associated with high mortality rates and, when treatment is possible, with complex recovery processes requiring prolonged convalescence until full recovery. In contrast, individuals admitted after falls into chimneys and similar structures, as well as those involved in vehicle collisions had the shortest rehabilitation periods, suggesting that these cases often involve less severe or more rapidly treatable conditions [19]. Mortality is generally high following vehicle collisions and in cases of severe injuries, similarly to other small mammals [53]. In the case of vehicle collisions, it can be assumed that squirrels surviving the impact are only mildly injured, resulting in shorter treatment times.

## 5. Conclusions

During the study period, an increasing trend in admissions of Eurasian red squirrels to rehabilitation centres was observed. The most frequently admitted category consisted of juveniles classified as orphaned, underdeveloped, or non-independent. More than half of the admitted squirrels were subsequently released back into the wild. This result indicates effective care provided by rehabilitation centres, even in the case of juveniles, whose rearing and subsequent adaptation can be challenging due to species-specific requirements.

Higher mortality was recorded in squirrels admitted following vehicle collisions. However, these individuals, together with those injured after falls into chimneys and similar structures, spent the shortest time in rehabilitation centres prior to release.

The results also highlight a significant impact of anthropogenic activity on the admission of Eurasian red squirrels to rehabilitation centres. Another important function of rehabilitation centres is therefore public education, particularly in distinguishing between truly injured animals and orphaned juveniles that require human assistance for survival.

## Figures and Tables

**Table 1:** Reasons for admission of Eurasian red squirrels to rehabilitation centres in the Czech Republic.

Reasons for Admissions	Definition
Juveniles—orphaned	Abandoned or underdeveloped juveniles, e.g., a juvenile found near its dead mother.
Juveniles from threatened nests	Juveniles from destroyed or threatened nests, e.g., an individual found near a destroyed nest/tree containing a nest.
Trauma	Injuries from causes other than traffic collisions or bites, e.g., fractures, contusions.
Vehicle collisions	An individual struck by a car and found near a road.
Animal attack	Injuries related to bite wounds, e.g., a squirrel reported after being bitten by a dog.
Infection, exhaustion	Infectious diseases (bacterial, viral, parasitic, or fungal) and exhaustion-related conditions, including prolonged starvation, diagnosed based on clinical signs such as coughing, diarrhoea, and poor body condition.
Unnecessarily captured	The individuals showed no signs of impairment.
Hand-rearing	Reared in a rehabilitation centre or other organisation and subsequently transferred to a rehabilitation centre.
Falls/Entrapment	Found after falling or trapped in chimneys, pits, or other unsecured openings.
Other	Other reasons for admission not listed above, e.g., poisoning or unknown.

**Table 2:** Outcome of Eurasian red squirrels admitted to rehabilitation centres in the Czech Republic.

Outcome	Definition
Release	Released back into the wild after recovery of attainment of independence
Death	Individuals that died during care in the rehabilitation centre (unassisted death).
Euthanasia	Euthanized by a veterinarian using veterinary medications.
Escape	Individuals that escaped from the rehabilitation centre, e.g., during handling.
Given to the other organisation/keeper	Individuals transferred to another organisation or qualified person.
In care	Individuals kept under care in rehabilitation centres at the time of submission of records to the Ministry of the Environment.
Other	Removed from records for reasons other than those listed above, unknown outcome.

**Table 3:** Number of Eurasian red squirrels admitted to rehabilitation centres in the Czech Republic between 2010 and 2020 by sex (*n* = 2252).

Sex	n	%	*p*
Female	1055	46.85	0.0002
Male	1197	53.15	

**Table 4:** Number of Eurasian red squirrels admitted to rehabilitation centres in the Czech Republic between 2010 and 2020 by reason for admission (*n* = 5365).

	Number of Admitted European Red Squirrels	
Reason for Admission	n	%
Juveniles—orphaned	3108	57.93 ^a^
Juveniles from threatened nests	954	17.78 ^b^
Trauma	350	6.52 ^c^
Vehicle collisions	306	5.70 ^c^
Animal attack	259	4.83 ^d^
Infection, exhaustion	141	2.63 ^e^
Unnecessarily captured	31	0.58 ^f^
Hand-rearing	31	0.58 ^f^
Falls/Entrapment	23	0.43 ^f^
Other	162	3.02 ^e^

^a–f^ Different letters within a column indicate a statistically significant differences (*p* < 0.05) in the numbers of admitted animals between the categories according to reason for admission.

**Table 5:** Number of Eurasian red squirrels admitted to rehabilitation centres in the Czech Republic between 2010 and 2020 by reason for removal from records (*n* = 5365).

Outcome	Number of Eurasian Red Squirrels Admitted to Rehabilitation Centres	
	n	%
Release	2827	52.69 ^a^
Death	1510	28.15 ^b^
Given to the other organisation/keeper	421	7.85 ^c^
Euthanasia	181	3.37 ^e^
Escape	17	0.32 ^f^
In care	173	3.22 ^e^
Other	236	4.40 ^d^

^a–f^ Different letters within a column indicate a statistically significant differences (*p* < 0.05) in the numbers of admitted animals between outcome categories.

**Table 6:** Distribution of outcomes of Eurasian red squirrels admitted to rehabilitation centres in the Czech Republic between 2010 and 2020 according to the reason for admission (*n* = 5365).

Reason for Admission	Outcome (% in Given Reason for Admission)						
	Release	Death	Given to the Other Organisation/Keeper	Euthanasia	Escape	In Care	Other
Juveniles—orphaned	59.17 ^a^	20.91 ^b^	9.52 ^c^	1.51 ^f^	0.19 ^g^	3.25 ^e^	5.44 ^d^
Juveniles from threatened nests	68.97 ^a^	17.09 ^b^	7.55 ^c^	0.94 ^e^	0.00 ^f^	3.98 ^d^	1.47 ^e^
Trauma	15.43 ^c^	48.86 ^a^	5.14 ^d^	21.71 ^b^	0.57 ^e^	4.57 ^d^	3.71 ^d^
Vehicle collisions	11.44 ^b^	72.88 ^a^	1.63 ^d,e^	7.19 ^b,c^	0.33 ^e^	2.61 ^d,e^	3.92 ^c,d^
Animal attack	25.48 ^b^	59.46 ^a^	3.47 ^c,d^	6.95 ^c^	0.39 ^e^	1.54 ^d^	2.70 ^d,e^
Infection, exhaustion	39.01 ^a^	51.06 ^a^	0.71 ^b,c^	4.26 ^b^	0.00 ^c^	3.55 ^b,c^	1.42 ^b,c^
Unnecessarily captured	61.29 ^a^	35.48 ^a^	3.23 ^b^	0.00 ^b^	0.00 ^b^	0.00 ^b^	0.00 ^b^
Hand-rearing	77.42 ^a^	9.68 ^b^	12.90 ^b^	0.00 ^b^	0.00 ^b^	0.00 ^b^	0.00 ^b^
Falls/Entrapment	73.91 ^a^	17.39 ^b^	0.00 ^b^	0.00 ^b^	4.35 ^b^	0.00 ^b^	4.35 ^b^
Other	37.04 ^a^	36.42 ^a^	9.26 ^c,d^	1.85 ^e^	3.70 ^d,e^	0.62 ^e^	11.11 ^c^

^a–g^ Different letters within a row indicate statistically significant differences (*p* < 0.05) in the proportions of individuals among outcome categories for a given reason for admission.

## Data Availability

Data for the analysis was obtained from the Ministry of the Environment of the Czech Republic. The datasets is available from the Figshare repository: Dataset. https://doi.org/10.6084/m9.figshare.32397978.
